# Collaborating eye to eye: Effects of workplace design on the perception of dominance of collaboration robots

**DOI:** 10.3389/frobt.2022.999308

**Published:** 2022-09-27

**Authors:** Alexander Arntz, Carolin Straßmann, Stefanie Völker, Sabrina C. Eimler

**Affiliations:** ^1^ Institute of Computer Science, University of Applied Sciences Ruhr West, Bottrop, Germany; ^2^ Institute of Mechanical Engineering, University of Applied Sciences Ruhr West, Mülheim an der Ruhr, Germany

**Keywords:** human-robot collaboration, non-verbal communication, dominance, dual-arm robots, industrial robots, online study, workplace ergonomics, human factors

## Abstract

The concept of Human-Robot Collaboration (HRC) describes innovative industrial work procedures, in which human staff works in close vicinity with robots on a shared task. Current HRC scenarios often deploy hand-guided robots or remote controls operated by the human collaboration partner. As HRC envisions active collaboration between both parties, ongoing research efforts aim to enhance the capabilities of industrial robots not only in the technical dimension but also in the robot’s socio-interactive features. Apart from enabling the robot to autonomously complete the respective shared task in conjunction with a human partner, one essential aspect lifted from the group collaboration among humans is the communication between both entities. State-of-the-art research has identified communication as a significant contributor to successful collaboration between humans and industrial robots. Non-verbal gestures have been shown to be contributing aspect in conveying the respective state of the robot during the collaboration procedure. Research indicates that, depending on the viewing perspective, the usage of non-verbal gestures in humans can impact the interpersonal attribution of certain characteristics. Applied to collaborative robots such as the Yumi IRB 14000, which is equipped with two arms, specifically to mimic human actions, the perception of the robots’ non-verbal behavior can affect the collaboration. Most important in this context are dominance emitting gestures by the robot that can reinforce negative attitudes towards robots, thus hampering the users’ willingness and effectiveness to collaborate with the robot. By using a 3 × 3 within-subjects design online study, we investigated the effect of dominance gestures (Akimbo, crossing arms, and large arm spread) working in a standing position with an average male height, working in a standing position with an average female height, and working in a seated position on the perception of dominance of the robot. Overall 115 participants (58 female and 57 male) with an average age of 23 years evaluated nine videos of the robot. Results indicated that all presented gestures affect a person’s perception of the robot in regards to its perceived characteristics and willingness to cooperate with the robot. The data also showed participants’ increased attribution of dominance based on the presented viewing perspective.

## 1 Introduction

For a long time in automated production processes, industrial robots and humans have performed their work strictly separated. Isolated by cages, industrial robots operated at safe distances from the personnel to prevent potentially hazardous situations (Hentout et al., 2019). A new paradigm in the industry has spawned a new category of industrial robots explicitly designed to collaborate with humans in close proximity. This approach bears an enormous labor multiplier for industrial production cycles, as the human worker and the industrial robot can complement each other in their respective skill set. Industrial robots are valued for their capability to lift heavy objects and their repeatability of precise tasks, whereas the human worker excels at intuition and experience-based decision making and reactivity towards procedure deviating circumstances (Ajoudani et al., 2018). In concept, both parties can benefit from each other through mutual assistance and form the basis for the subject and research discipline of Human-Robot Collaboration (HRC).

While dedicated collaboration robots can come in many forms depending on their respective specializations Buxbaum et al. (2020), the configuration of a dual-arm setup for collaboration robots promises to mimic the actions and capabilities of the human collaboration partner best (Kirschner et al., 2016). This is based on the assumption that dual-arm robots can mirror the capability of humans to operate as bilateral capable manipulators. Apart from projected advantages for the collaboration procedure itself, such as enhanced coordination capabilities, the physical representation of a dual-arm robot allows for extensive gesture-based communication. Although incapable of mirroring the body language capabilities of modern androids, industrial dual-arm robots can express some gestures that resemble human-like postures. Therefore, dual-arm collaboration robots could be outfitted to signal and adjust a variety of different gestures in accordance with the current collaboration context. Prior studies regarding the interaction with robots revealed a significant benefit of collaboration robots equipped with gesture-based communication combined with other information interfaces during shared task scenarios with a human partner (Arntz et al., 2021a; Bremner and Leonards, 2016). Ranging from subjective benefits such as reduced stress and objective benefits regarding the production quantity, it can be assumed that dual-arm robots can be enhanced in their collaboration effectiveness through communication as well. However, while prior studies used established gestures for industrial robots represented by a single-arm setup (Ende et al., 2011), the evaluation of dual-arm gestures for collaboration settings with humans still needs further investigation.

Since research in the domain of Human-Robot Interaction indicates the multi-layered complexity in regards to the information that is conveyed by robots using human-like gestures and body language (Riek et al., 2010; McColl and Nejat, 2014), it is paramount to explore the respective perceptions that users gain from dual-collaboration robots equipped with gesture-based communication. The goal is to incrementally over a series of studies evaluate a library of different gestures and investigate their respective impression on users to sort out unfit gestures for dual-arm robots that might compromise the collaboration experience. To achieve this, the study presented in this work tested three distinct gestures for dominance derived from the works of Straßmann et al. that investigated the effects of different nonverbal gestures of virtual agents regarding the perception of dominance (Straßmann et al., 2016). Since a substantial number of people in Western societies uphold several misconceptions and fears about robots in a working context, i.e., predominantly the fear of being replaced (Union, 2014), it is important to investigate gesture-based communications for robots that do not reinforce the notion of being dominated. Another aspect that is essential for an individual’s perception of the threat and dominance of other humans and robots is the individual’s spatial perspective and position (Re et al., 2014). With regard to the design of workplace ergonomics for HRC setups, it is important to investigate potential multipliers for the unwanted perception of dominance by the collaborative robot. To consider this, the study presented the robot from multiple perspectives based on the height of the human operator that is exposed to the gestures of the dual-arm robot, resulting in the three conditions using the average height of females, males, and a sitting position. With regards to the ergonomics of HRC workplace arrangements, it is anticipated that the view to which the user is exposed to the robot can affect the way the robot is perceived (Stanton and Stevens, 2017).

The subsequent sections outline the theoretical foundations of the hypothesis and research questions guiding this work. After that, the methodological details of the empirical study are presented. In the end, results are reported followed by a discussion of results and limitations.

## 2 Related work

Akin to the group collaboration among humans, research indicates that the effective collaboration between humans and robots requires the exchange of information to facilitate coordination of the current state of each entity and the handled tasks (Arntz et al., 2021b; Hentout et al., 2019). The medium to convey the necessary information can be delegated to a wide array of possible channels, i.e., speech, text, light signals, or gestures (Arntz et al., 2020a). Since the collaboration of humans and robots follows an embodied form of interaction between the two parties, it is expected that the usage of gestures is naturally embedded in people’s social communication while collaborating with the robot (Hentout et al., 2019). Research has shown that robots specifically tasked with collaboration procedures induce higher expectations regarding the robots’ capability to respect social norms such as proxemics and gestures that suit the current context (Mumm and Mutlu, 2011). However, prior studies on the usage of gestures for industrial robots did not replicate gestures directly adapted from human posture (Ende et al., 2011). The reason is the difficult direct translation of human-like gestures onto the various non-humanoid representations of industrial robots. However, the application of human-like characteristics to industrial robots can be found in some attempts from robot manufacturers to provide human personnel with an anchor to facilitate peoples’ willingness for interaction. A common example of this is the implementation of human-like facial expressions on the Baxter robot (Si and McDaniel, 2016). However, since the goal of the application of human characteristics is to elevate people’s willingness to collaborate with the robot and reduce unfavorable prejudices, it is paramount for the robot to emit a non-threatening and dominating presence (Buxbaum et al., 2020).

Research regarding communication among humans has shown, that across cultures gestures and body language can be reinforced based on the respective hierarchy level an individual is perceived to have (Chase and Lindquist, 2009). The authors argue with the prior-attributes hypothesis which postulates that the attribution of dominance can be affected by behavioral characteristics such as aggressiveness but also the physical representation such as height Chase and Lindquist (2009). The posture of an individual and the relational perspective of the observer can affect the observers’ perception regarding the dominance and other characteristics of the respective person (Marshall et al., 2020). Human-Robot Interaction studies have shown that some of the associations gained from human body language can be applied to social robots with humanoid representations as well (Beck et al., 2012; McColl and Nejat, 2014). According to the research of Chung-En, humanoid robots outfitted with a smiling face and accompanied by adequate body language evoked similar perceptions of interpersonal warmth across all ages and genders (Chung-En, 2018). While collaborative robots deployed in industrial settings do not follow a human-like representation as robots deployed in social contexts, it can be argued that the Yumi IRB 14000 with its dual-arm setup designed to mimic human action can evoke a more human-like association (Kirschner et al., 2016). This is based on the works of Lee et al., where the authors designed a dual-arm robot following a close resembling structure to the Yumi IRB 14000, with the goal of a biologically inspired anthropomorphic representation (Lee et al., 2017). It can be argued the attribution of human characteristics can be applied to dual-arm collaboration robots with their anthropomorphic resemblance. Therefore the perspective-based reinforcement of characteristics such as dominance should be applicable as well.

Another important aspect is the effect the perspective has on the respective gestures. Since the interpretation of body language does not follow the same precision as direct messaging, gestures designed to convey a certain state can be interpreted differently by industrial staff based on their respective position, thus jeopardizing the intent of the gesture. Another aspect that is crucial for the upcoming HRC scenario is the ergonomics of the workplace setup. Industrial staff can collaborate with the robotic partner in a seated or standing position. Therefore it is of interest how the shift in perspective affects the impression gained from the gestures made by the robot. Since a variety of perceptions regarding the dominance of an entity exists across different demographics such as age groups (Rosenthal-von der Pütten et al., 2019), it is of interest if there are gender-specific differences in the dominance-related perception of the robot. This is grounded in the works of Sokolov et al., which indicate that women tend to read body language, especially hostile gestures more effectively than men (Sokolov et al., 2011).

### 2.1 Hypothesis and research questions

Based on the theoretical work outlined before one hypothesis (H1) and two research questions (RQ1 and RQ2) were deduced focusing on the attributions made to the robot arm’s dominance gestures and considering the users’ workplace configuration (average female and male height and a sitting setup) and gender.

• H1: The viewing perspective has an effect on participants perceived dominance of the dual-arm robot.
• RQ1: How does the human’s viewing perspective affect the perception of the robot’s gestures?
• RQ2: What are the differences in perception between both genders?

## 3 Methods

The study used an HRC workplace arrangement containing the Yumi IRB 14000 dual-arm robot within an industrial background scene for immersion purposes (ABB, 2015). The online experiment followed a within design comprised of a series of twelve first-person perspective videos.

### 3.1 Sample

The sample consisted of 115 participants (female = 58, male = 57) with an average age of *M* = 24.47 (*SD* = 6.26). Only *N* = 5 participants indicated to have worked with the robot before, while *N* = 10 indicated to have seen the dual-arm robot in a real environment.

### 3.2 Measures and procedure

Self-reported data collected through an online questionnaire were used to investigate the postulated hypothesis and research question. Presented through the online platform SoSciSurvey Survey (2022), participants were asked to fill out a questionnaire that was formulated in German. Items derived from English language sources were translated independently by two researchers to guarantee a proper translation. The landing page introduced participants to all information required to provide informed consent. After agreeing to take part, participants were informed about data protection handling and asked to generate a code allowing the anonymous deletion of their data after the study if they wished so. After that their age and gender were collected, which was necessary to explore RQ2. This was followed by a short briefing regarding the stimulus material explaining that participants would see 12 short videos of an industrial robot and be asked to answer the question in an undisturbed environment where they could follow the videos with full attention. Also, they were informed that they would not need audio and could also use their smartphone but would need to adjust the display size.

After that, participants were exposed to the videos. Each of the videos was accompanied by a set of questions consisting of a) a list of 17 items of a semantic differential (5-point scale) and b) one item with a Kunin-scale (7-point scale). To measure anthropomorphism (conscious—unconscious (inverse) and artificial—lifelike), animacy (stagnant—lively and artificial—lifelike), and likability (unpleasant—pleasant, dislike—like), a selection of 2 items each from the Godspeed subscales (Bartneck et al., 2008) were used. The Cronbach’s alpha values of the subscale anthropomorphism (*α* = .402) and animacy (*α* = .558) are rather low and the internal consistency is therefore threatened. However, this can be explained by the reduced number of item (2 items) that was necessary due to the repeated measures approach and the overall length of the questionnaire. Since these variables are of high interest for the research aim of this study, the measures are used despite the low internal consistency. For the subscale likability the internal consistency was acceptable (*α* = .781).

Along the interpersonal circumplex (Orford, 1994) the perceived dominance (dominant—submissive) and hostility (hostile—friendly) of the robot were measured. Additionally, two self-generated single items were used to measure the perceived cooperativeness (uncooperative—cooperative) and threat (threatening—harmless) of the robot. Moreover, the feelings during an imagined collaboration with the robot were measured with a single item (“If the robot behaved as it does in the video shown: How comfortable are you with the thought of working together with the robot?”) rated on a Kunin-scale (7-point scale).The order of the videos was shuffled at random for each of the participants to prevent the formation of participants answering the items along an emerging pattern. In the end, participants were asked if they are familiar with the presented robot and if they ever collaborated with the Yumi IRB 14000 robot. Also, they were asked if there had been technical problems and given the chance to give feedback on positive or negative aspects they noticed about the study before being fully debriefed.

### 3.3 Stimulus material

The stimulus material consisted of twelve videos in a 3 × 3 setup with the three gestures dubbed Akimbo, crossing arms and large arm spread, and the respective three height positions derived from the global average female height of 159 cm (Roser et al., 2022), the global average male height of 177 cm and a sitting position (133 cm above the ground) in front of the Yumi IRB 14000 dual-arm robot manufactured by ABB (ABB, 2015). The dual-arm robot was presented within an industrial background and the appropriate soundscape to facilitate the HRC context of the study. On average, the video stimulus material presented the respective gesture in a fifteen seconds time frame.

#### 3.3.1 Akimbo

**FIGURE 1:**
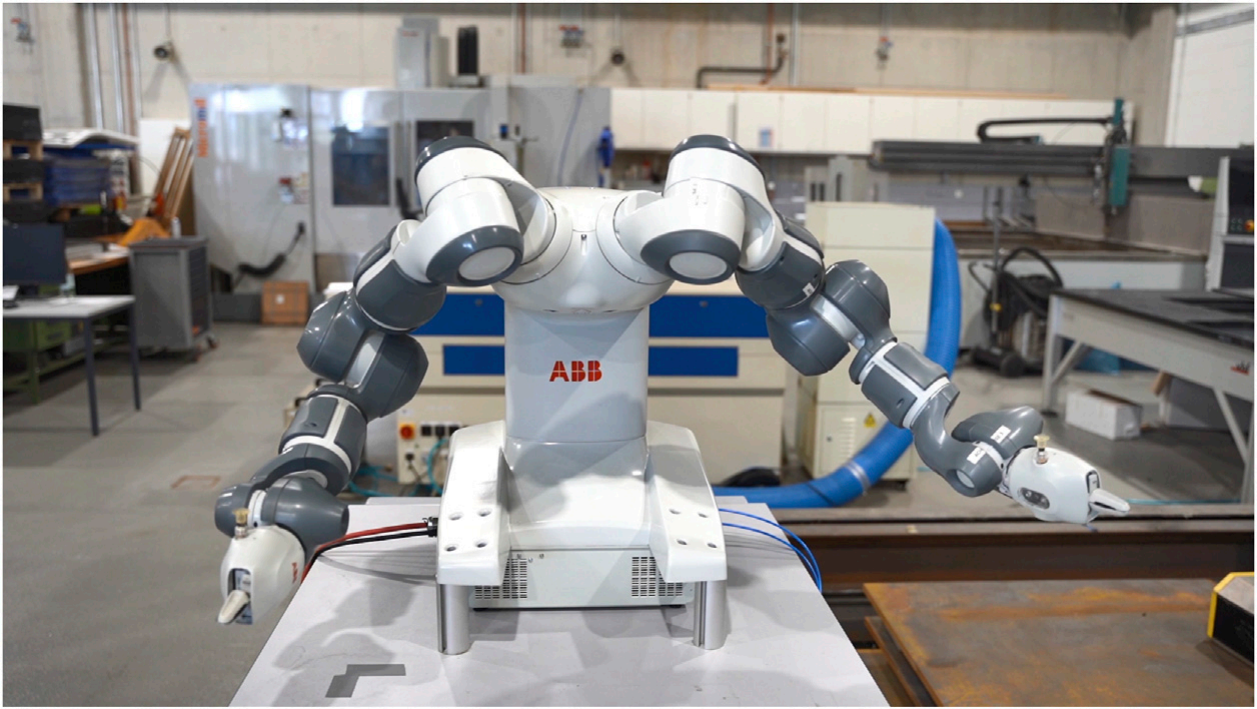
The Akimbo gesture where both arms of the robot are placed on the “hips” of the dual-arm robot.

The placement of the arms on the hips, which is referred to as Akimbo, is a readiness stance that is regarded as a confident posture among humans (Ball and Breese, 2000). This was difficult to recreate with the robot since the Yumi IRB 14000 does not have humanoid characteristics, thus no representation of hips was present (Figure 1). As a substitute, the extension of the robots’ supporting surface was used a reference point for the placement of the Akimbo gesture to imitate the human posture as close as possible. The dual-arm configuration started from the robot’s initial position and maintained a steady upwards trajectory before shifting position midway towards the designated hips of the robot while widening the elbows of the arms outwards to emphasize the dominant position.

#### 3.3.2 Crossing arms

**FIGURE 2:**
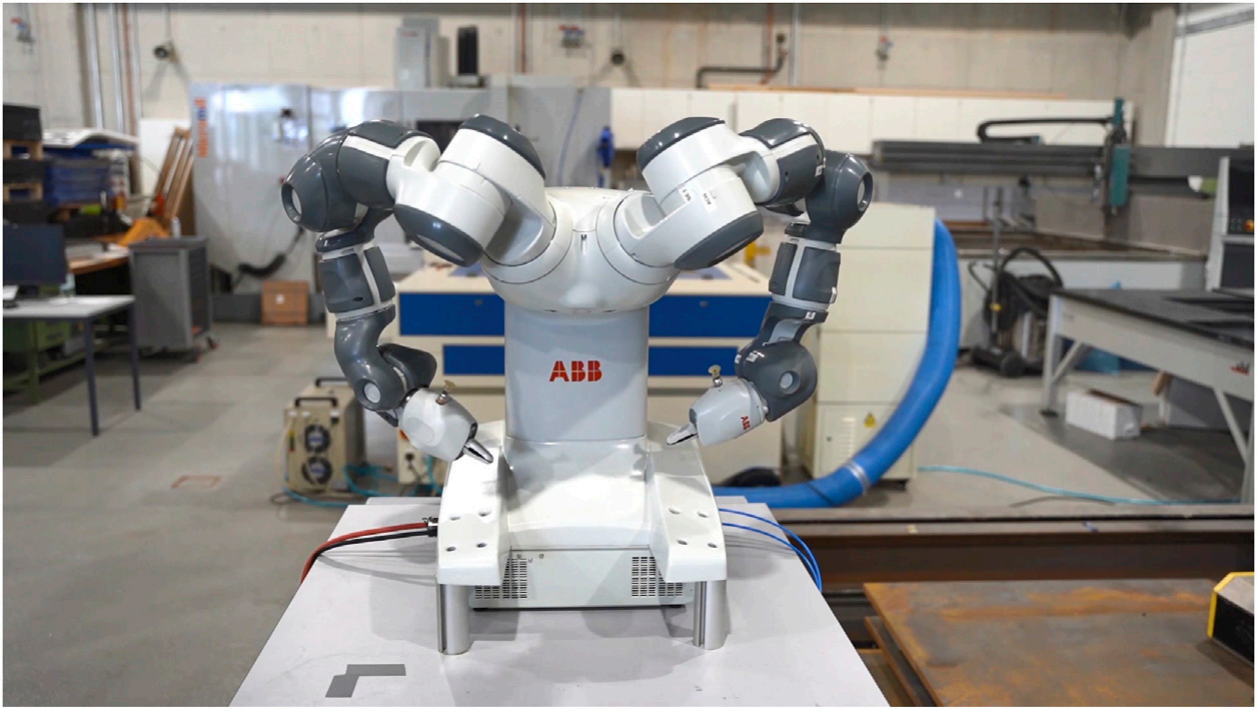
The crossing arm gesture as illustrated in the stimulus material.

In Human-Human Interaction the crossing of arms is usually interpreted as a defiant or defensive posture that can indicate that the respective person is denying or disagreeing with the current circumstance or situation (Danbom, 2008). This gesture was chosen because it can indicate stubbornness, uncooperativeness, and dominance, all characteristics that can be detrimental to the collaboration effectiveness between two parties. The crossing arms gesture was designed to imitate the crossing arm posture of a human being based on the works of Straßmann et al. (Straßmann et al., 2016). While the human expression of this body language commonly involves direct contact of both arms while they cross, the direct translation of this gesture on the Yumi IRB 14000 is not possible (cf. Section 5.1 Limitations). To approximate this gesture as close as possible to its human counterpart, the robot started from the initial neutral position to a posture where arms were aligned vertical towards the central body, then the joints that can be seen as an analog of shoulders and the elbows rotated inwards so that each arm assumed a trajectory that resulted in a parallel position to the robots’ body pointing to the respective opposite position. Although, the robot does not cross its arms in this stance, from the perspective of the human operator the impression of the crossing arm gesture can be made (Figure 2).

#### 3.3.3 Large arm spread

**FIGURE 3:**
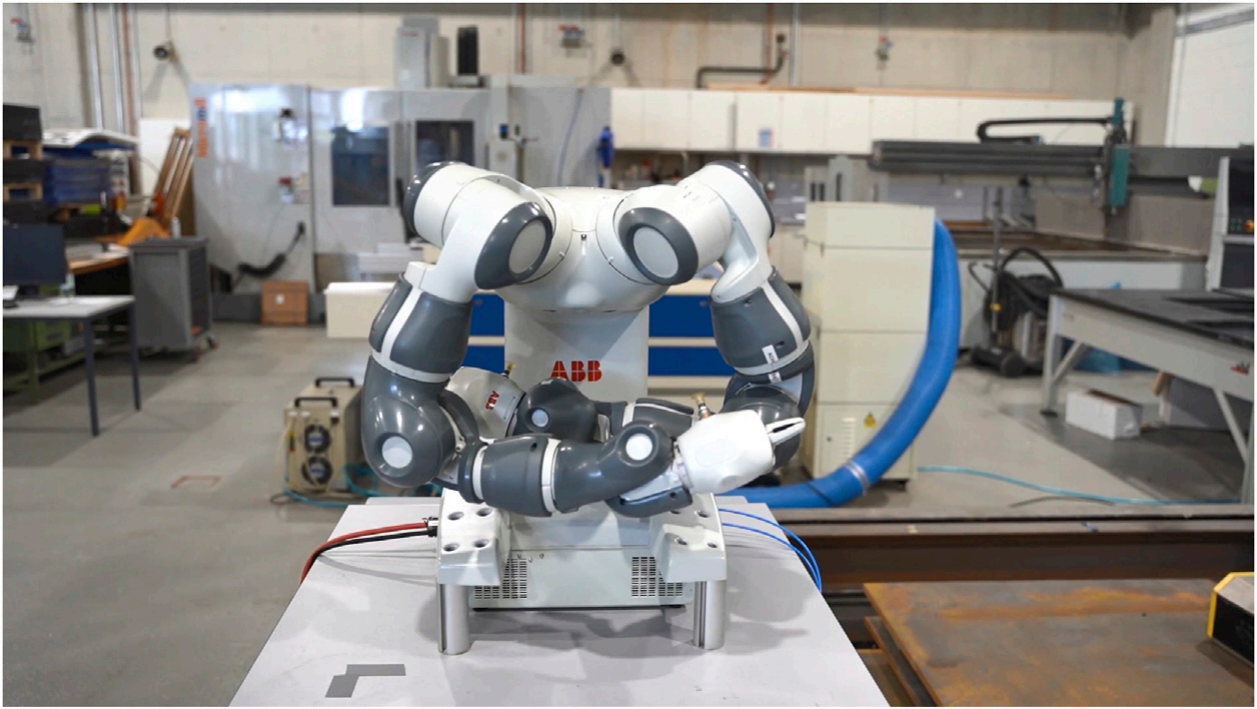
The large arm spread presented by the Yumi IRB 14000 dual-arm robot.

Robotic arms that spread themselves in the direction of the human operator and violate the proxemics of the respective individual are often considered threatening (Arntz et al., 2020b). Based on the prior works by Straßmann et al. (Straßmann et al., 2016), the large arm spread was conceptualized as a threatening gesture, where the posture of the robot indicates that the robot claimed to occupy the available space for itself. To realize this gesture both arms of the Yumi IRB 14000 start out in their respective neutral position. Here, both arms were retracted in an upright position and aligned with the body of the robot. At first, both arms moved simultaneously downward and forwards in the direction of the observer. After reaching the middle of the body of the robot, the trajectory of both arms diverted to an outward position, resulting in the final posture of the large arm spread (Figure 3).

## 4 Results

To investigate the above-mentioned hypotheses and research questions, data collected *via* the online study were analyzed using multiple mixed-measures ANOVAs with the repeated-measures variables gesture (large arm spread, Akimbo pose, and crossing arms) and viewing perspective (sitting, standing male perspective, and standing female perspective), and participants’ gender (binary: male and female) as between-subjects factor. For the repeated measures the assumption of sphericity was checked using Mauchly’s test, a statistical procedure used to validate a repeated measures analysis of variance (ANOVA) (Mauchly, 1940). If this assumption was violated, corrected results are reported. Since Greenhouse-Geisser Epsilon was above 0.75 in all cases (Geisser and Greenhouse, 1958), the Huyn-Feldt correction was used. Significant effects are further investigated with a post-hoc test using Bonferroni correction. As participants were not forced to answer all items in the questionnaire, and the sample sizes of the analyses vary between the dependent variables. However, in each of the cases, the full data set comprised at least 50 male and 50 female subjects. Subsequently, the results of these analyses are reported for all dependent variables.

### 4.1 Anthropomorphism

A main effect of the gesture (*F*(2,206) = 11.85, *p* < .001, *par. n* ^2^ = .10) and viewing perspective (*F*(1.93, 199.09) = 3.30, *p* = .041, *par. n* ^2^ = .03) in the perceived anthropomorphism of the robot occurred. To investigate the differences between the robot’s gestures, post-hoc analyses with Bonferroni correction were used. Results indicate that the Akimbo pose (*M* = 2.27, *SE* = 0.08) was perceived as less anthropomorphic than the large gesture (*M* = 2.50, *SE* = 0.09, *p* = .016) and a robot that crosses its arms (*M* = 2.64, *SE* = 0.18, *p* < .001). The effect of the viewing perspective in the post-hoc analyses, where the perceived anthropomorphism did not differ between the three viewing perspectives, disappeared. No significant interaction between gesture and viewing perspective was found. Participants’ gender had no effect on the perceived anthropomorphism of the robot (*F*(1,103) = 1.89, *p* = .171, *par. n* ^2^ = .02) nor are there any significant interaction effects between gender and the repeated-measure variables. Detailed results of the mixed-measures ANOVA are reported in Table 1.

**TABLE 1:** Results of the mixed-measures ANOVA for the perceived anthropomorphism of the robot.

Predictor	*dfNum*	*dfDen*	*Epsilon*	*SSNum*	*SSDen*	*F*	*p*	*η* ^2^
Gesture	2	206	0.96	11.16	0.94	11.85	< .001	.10
Viewing Perspective	1.93	199.09	0.94	1.26	0.38	3.30	.041	.03
Gesture X Viewing Perspective	4	412	0.95	0.32	0.30	1.05	.382	.01
Gesture X Gender	2	206		0.46	0.94	0.49	.611	.01
Viewing Perspective X Gender	1.93	199.09		0.07	0.38	0.18	.830	.00
Gesture X Viewing Perspective X Gender	4	412		0.36	0.30	1.18	.318	.01

### 4.2 Animacy

The robot’s perceived animacy was only affected by the displayed gesture of the robot (*F*(1.94,211.68) = 23.31, *p* < .001, *par. n* ^2^ = .18); no effect of the viewing perspective and no interaction effect between both variables occurred. The post-hoc analyses revealed that a robot showing the Akimbo pose (*M* = 2.46, *SE* = 0.07) was rated with lower animacy than one presenting the large arm spread gesture (*M* = 2.95, *SE* = 0.07, *p* < .001) and a robot that uses the crossing arm gesture (*M* = 2.85, *SE* = 0.07, *p* < .001). Additionally, the participants’ gender had no significant effect on the perception of the robot’s animacy (*F*(1,109) = 3.17, *p* = .078, *par. n* ^2^ = .03) and no interaction effects with participants gender were found. See Table 2 for the results of the mixed-measures ANOVA.

**TABLE 2:** Results of the mixed-measures ANOVA for the perceived animacy of the robot.

Predictor	*dfNum*	*dfDen*	*Epsilon*	*SSNum*	*SSDen*	*F*	*p*	*η* ^2^
Gesture	1.94	211.68	0.95	23.52	1.01	23.31	< .001	.18
Viewing Perspective	1.62	176.29	0.79	0.00	0.46	0.01	.982	.00
Gesture X Viewing Perspective	3.50	381.43	0.84	0.29	0.32	0.90	.452	.00
Gesture X Gender	1.94	211.68		0.62	1.01	0.62	.536	.01
Viewing Perspective X Gender	1.62	176.29		0.13	0.46	0.29	.702	.00
Gesture X Viewing Perspective X Gender	3.50	381.43		0.27	0.32	0.86	.477	.01

### 4.3 Likability

The mixed-measures ANOVA revealed a significant main effect for the gesture on the perceived likability of the robot (*F*(2,210) = 7.98, *p* < .001, *par. n* ^2^ = .07), but no significant main effect for the viewing perspective and no interaction effect. According to the post-hoc results, the robot is perceived as more likable when it presents large arm spread gesture (*M* = 3.51, *SE* = 0.07) compared to the Akimbo pose (*M* = 3.19, *SE* = 0.08, *p* = .004) and the crossing arm gesture (*M* = 3.16, *SE* = 0.09, *p* = .003). Again, there were no interaction effects with participant’s gender and the perceived likability rating was in general not affected by participants’ gender, *F*(1,105) = 0.00, *p* = .982, *par. n* ^2^ = .00. Please consult Table 3 for the values of the mixed-measures ANOVA.

**TABLE 3:** Results of the mixed-measures ANOVA for the perceived likability of the robot.

Predictor	*dfNum*	*dfDen*	*Epsilon*	*SSNum*	*SSDen*	*F*	*p*	*η* ^2^
Gesture	2	210	0.99	11.71	1.47	7.98	< .001	.07
Viewing Perspective	2	210	0.96	0.70	0.38	1.84	.162	.02
Gesture X Viewing Perspective	4	420	0.98	0.34	0.39	0.87	.483	.01
Gesture X Gender	2	210		1.34	1.47	0.91	.402	.01
Viewing Perspective X Gender	2	210		0.27	0.38	0.72	.487	.01
Gesture X Viewing Perspective X Gender	4	420		0.65	0.39	1.68	.153	.02

### 4.4 Dominance

The perceived dominance of the robot is significantly affected by the expressed gesture (*F*(2,216) = 4.47, *p* = .013, *par. n* ^2^ = .04) and the viewing perspective (*F*(2,216) = 4.96, *p* = .008, *par. n* ^2^ = .04), but no significant interaction effect occurred. Post-hoc results show that the crossing arm gesture (*M* = 2.93, *SE* = 0.08) is perceived as more dominant than the Akimbo pose (*M* = 3.18, *SE* = 0.07, *p* = .023). Here higher values indicate lower dominance and higher submissiveness all descriptive values can be found in Table 4. Moreover, a significant difference between the sitting and male standing perspective was revealed by the post-hoc analyses: The robot is perceived as more dominant from the sitting perspective (*M* = 2.98, *SE* = 0.06) compared to the male standing perspective (*M* = 3.18, *SE* = 0.06, *p* = .008). The female perspective (*M* = 3.12, *SE* = 0.07) did not differ in perceived dominance from the male and sitting viewpoint. The participants’ gender did not affect the dominance perception of the robot (*F*(1,108) = 2.74, *p* = .101, *par. n* ^2^ = .03) and there were also no significant interaction effects of the gender with the other two independent variables. See Table 5 for details.

**TABLE 4:** Descriptive values of the three different gestures for all dependent variables.

	Large arm spread		Akimbo		Crossing arms	
	*M*	*SE*	*M*	*SE*	*M*	*SE*
Anthropomorphism (high values indicate high anthropomorphism)	2.50	0.09	2.27	0.08	2.64	0.10
Animacy (high values indicate high animacy)	2.95	0.07	2.46	0.07	2.85	0.07
Likability (high values indicate high likability)	3.51	0.07	3.19	0.08	3.16	0.09
Dominance (high values indicate low dominance)	3.15	0.06	3.18	0.07	2.93	0.08
Hostility (high values indicate low hostility)	3.60	0.07	3.26	0.07	3.21	0.09
cooperativeness (high values indicate high cooperativeness)	3.63	0.08	3.33	0.08	3.07	0.09
Threat (high values indicate low threatenting)	3.79	0.07	3.66	0.09	3.41	0.10
Imagined collaboration with the robot (high values indicate positive emotions to cooperate)	5.24	0.10	4.95	0.13	4.88	0.12

**TABLE 5:** Results of the mixed-measures ANOVA for the perceived dominance of the robot.

Predictor	*dfNum*	*dfDen*	*Epsilon*	*SSNum*	*SSDen*	*F*	*p*	*η* ^2^
Gesture	2	216	0.98	5.94	1.33	4.47	.013	.04
Viewing Perspective	2	216	1.00	3.29	0.67	4.96	.008	.04
Gesture X Viewing Perspective	3.84	414.29	0.91	1.04	0.66	1.59	.180	.01
Gesture X Gender	2	216	—	2.19	1.33	1.65	.195	.02
Viewing Perspective X Gender	2	216	—	0.51	0.67	0.77	.464	.01
Gesture X Viewing Perspective X Gender	3.84	414.29	—	0.53	0.66	0.81	.513	.01

### 4.5 Hostility

The perceived hostility of the robot is significantly affected by the expressed gesture (*F*(2,220) = 10.04, *p* < .001, *par. n* ^2^ = .08) and the viewing perspective (*F*(2,220) = 3.95, *p* = .021, *par. n* ^2^ = .04), but no significant interaction effect occurred. According to the post-hoc results, the robot is perceived as more friendly and less hostile when it presents the large arm spread gesture (*M* = 3.60, *SE* = 0.07) compared to the Akimbo pose (*M* = 3.26, *SE* = 0.07, *p* = .001) and the crossing arm gesture (*M* = 3.21, *SE* = 0.09, *p* = .001). Here higher values indicate a higher ascription of friendliness, while lower values indicate attributions towards more hostility. Moreover, a significant difference between the sitting and male standing perspective was revealed by the post-hoc analyses: The robot is perceived as less friendly from the sitting perspective (*M* = 3.29, *SE* = 0.06) compared to the male standing perspective (*M* = 3.44, *SE* = 0.06, *p* = .034) The participants’ gender had no effect on the perceived hostility/friendliness of the robot, *F*(1,110) = 0.12, *p* = .725, *par. n* ^2^ = .00 and there was no significant interaction between the participants’ gender and the gesture or viewing perspective. Consult Table 6 for all values of the mixed-measures ANOVA.

**TABLE 6:** Results of the mixed-measures ANOVA for the perceived hostility of the robot.

Predictor	*dfNum*	*dfDen*	*Epsilon*	*SSNum*	*SSDen*	*F*	*p*	*η* ^2^
Gesture	2	220	0.98	15.35	1.53	10.04	< .001	.08
Viewing Perspective	2	220	0.97	1.88	0.48	3.95	.021	.04
Gesture X Viewing Perspective	3.80	418.19	0.91	0.95	0.52	1.82	.128	.02
Gesture X Gender	2	220	—	1.36	1.53	0.89	.413	.01
Viewing Perspective X Gender	2	220	—	0.05	0.48	0.10	.903	.00
Gesture X Viewing Perspective X Gender	3.80	418.19	—	0.16	0.52	0.30	.866	.00

### 4.6 Cooperativeness

The perceived cooperativeness of the robot is significantly affected by the expressed gesture (*F*(2,220) = 14.48, *p* < .001, *par. n* ^2^ = .12). No significant main effect for the viewing perspective and no interaction effect occurred. According to the post-hoc results, the robot is perceived as cooperative when it presents the large arm spread gesture (*M* = 3.63, *SE* = 0.08) compared to the Akimbo pose (*M* = 3.33, *SE* = 0.08, *p* = .008) and the crossing arm gesture (*M* = 3.07, *SE* = 0.09, *p* < .001). Also, the Akimbo pose is associated with significantly higher values of cooperativeness than the crossing arm gesture (*p* = .039). Here higher values indicate higher attributed levels of cooperativeness. Again, no interaction effects of participants’ gender and the other two independent variables occurred (see Table 7) and gender had no effect on the general perception of the robot’s cooperativeness, *F*(1,110) = 0.30, *p* = .583, *par. n* ^2^ = .00.

**TABLE 7:** Results of the mixed-measures ANOVA for the perceived cooperativity of the robot.

Predictor	*dfNum*	*dfDen*	*Epsilon*	*SSNum*	*SSDen*	*F*	*p*	*η* ^2^
Gesture	2	220	0.97	26.35	1.82	14.48	< .001	.12
Viewing Perspective	2	220	0.98	0.17	0.65	0.62	.772	.00
Gesture X Viewing Perspective	4	440	0.97	0.32	0.61	0.52	.719	.01
Gesture X Gender	2	220	—	1.88	1.82	1.03	.358	.01
Viewing Perspective X Gender	2	220	—	0.06	0.65	0.10	.908	.00
Gesture X Viewing Perspective X Gender	4	440	—	1.00	0.61	1.64	.163	.02

### 4.7 Threat

The perceived threat of the robot is significantly affected by the expressed gesture (*F*(2,220) = 7.62, *p* = .001, *par. n* ^2^ = .07). No significant main effect for the viewing perspective and no interaction effect occurred. Post-hoc tests show that the robot is perceived as more harmless when it presents the large arm spread gesture (*M* = 3.79, *SE* = 0.07) compared to the crossing arm gesture (*M* = 3.41, *SE* = 0.10, *p* = .001) and the Akimbo pose (*M* = 3.66, *SE* = 0.09, *p* = .030). Higher values indicate lower perceived threat and higher harmlessness. The perceived threat of the robot was not affected by participants’ gender, (*F*(1,110) = 0.22, *p* = .641, *par. n* ^2^ = .00). In addition, the effect of the gesture and viewing perspective on the perceived threat was also not affected by gender (see Table 8).

**TABLE 8:** Results of the mixed-measures ANOVA for the perceived threat of the robot.

Predictor	*dfNum*	*dfDen*	*Epsilon*	*SSNum*	*SSDen*	*F*	*p*	*η* ^2^
Gesture	2	220	0.98	12.19	1.60	7.62	< .001	.07
Viewing Perspective	2	220	0.96	0.96	0.64	1.50	.226	.01
Gesture X Viewing Perspective	3.83	420.84	0.91	0.40	0.78	0.52	.713	.01
Gesture X Gender	2	220	—	1.84	1.60	1.15	.318	.01
Viewing Perspective X Gender	2	220	—	0.06	0.64	0.09	.918	.00
Gesture X Viewing Perspective X Gender	3.83	420.84	—	0.27	0.78	0.34	.840	.00

### 4.8 Imagined collaboration with the robot

The imagination to collaborate with the robot is significantly affected by the gesture (*F*(2,220) = 5.96, *p* = .003, *par. n* ^2^ = .05). No significant main effect for the viewing perspective and no interaction effect occurred. Post-hoc tests show that participants feel more positive to collaborate with the robot when the robot shows the large arm spread gesture (*M* = 5.24, *SE* = 0.10) compared to the Akimbo pose (*M* = 4.95, *SE* = 0.13, *p* = .038) and the crossing arm gesture (*M* = 4.88, *SE* = 0.12, *p* = .006). Again no main effect of participants’ gender as between factor (*F*(1,110) = 0.00, *p* = .984, *par. n* ^2^ = .00) and no interaction effects (consult Table 9) with the other two variables on the imagined collaboration with the robot was found.

**TABLE 9:** Results of the mixed-measures ANOVA for the imagined collaboration with the robot.

Predictor	*dfNum*	*dfDen*	*Epsilon*	*SSNum*	*SSDen*	*F*	*p*	*η* ^2^
Gesture	2	220	0.98	12.29	2.06	5.96	.003	.05
Viewing Perspective	2	220	0.99	0.29	0.45	0.65	.524	.01
Gesture X Viewing Perspective	3.62	397.77	0.86	0.30	0.60	0.50	.715	.01
Gesture X Gender	2	220		1.15	2.06	0.56	.573	.01
Viewing Perspective X Gender	2	220		0.34	0.45	0.76	.467	.01
Gesture X Viewing Perspective X Gender	3.62	397.77		0.40	0.60	0.67	.601	.01

## 5 Summary of the results

To obtain the pattern that underlays the above-presented results, Table 10 presents the significant effects of all independent variables and their interaction effects on the measured dependent variables. Overall, results indicate that the gestures conducted by the robot and the respective viewing perspective affected the perceived dominance of the robot. The robot is perceived as the most dominant in the sitting perspective whereas the male perspective resulted in the lowest attribution of dominance out of the three perspectives. This coincides with the theoretical outline presented in Section 2 and renders H1 supported.

**TABLE 10:** Overview of the significant differences for all dependent variables. High values indicate a high degree of the respective attribute category.

	Gesture	Viewing perspective	Gesture X viewing perspective	Gender	Gesture X gender	Viewing perspective X gender	Gesture X ViewingPerspective X gender
Anthropomorphism	Akimbo < Large	No effect	No effect	No effect	No effect	No effect	No effect
	Akimbo < Crossing arms						
Animacy	Akimbo < Large	No effect	No effect	No effect	No effect	No effect	No effect
	Akimbo < Crossing arms						
Likability	Large > Akimbo	No effect	No effect	No effect	No effect	No effect	No effect
	Large > Crossing arms						
Dominance	Crossing arms < Akimbo	Sitting < Standing male	No effect	No effect	No effect	No effect	No effect
Hostility	Large > Akimbo	Sitting < Standing male	No effect	No effect	No effect	No effect	No effect
	Large > Crossing arms						
Cooperativeness	Large > Akimbo	No effect	No effect	No effect	No effect	No effect	No effect
	Large > Crossing arms						
Threat	Crossing arms < Large	No effect	No effect	No effect	No effect	No effect	No effect
	Crossing arms < Akimbo						
Imagined collaboration with the robot	Large > Akimbo	No effect	No effect	No effect	No effect	No effect	No effect
	Large > Crossing arms						

For RQ1, results indicate that the attribution of threat by the robot is higher in the large arm spread gesture. This contradicts the order of perceived dominance found in the effects of the other two gestures. It can be argued that the posture of large arms spread by the robot can elicit a more threatening perception as the robot’s size and reach of the arms are seen as a potential hazard that can provoke accidents due to unintended collisions with the robot.

Regarding RQ2, results indicated that the gender of the participants did not affect the perception of the robot. In addition to the perception gained from the respective gestures, no significant differences regarding the gender of the participant emerged from the different viewing perspectives. This implies that in terms of workplace ergonomics, gestures can be utilized independent of the staff’s gender and viewing perspective without creating detrimental effects for a specific condition that hampers the collaboration procedure. This renders H1 supported. Regarding the second research question (RQ2), which addressed potential differences in gender due to the diverging average height. Results indicated that the gender of the participants did not affect the perception of the robot. In addition to that, no influence on the effect of the different gestures viewing perspectives has been found.

### 5.1 Limitations

To contextualize the results it is essential to address the limitations of this study. A major limitation is that the questionnaire did not ask participants about their actual body height but rather assumed the assigned perspective based on the stated gender of the participants. It is advised that a future study should be conceptualized as a lab study where the actual body height of the participants is considered. Since virtual reality has meanwhile become a valuable methodological approach to studies mimicking future workplace scenarios, see e.g. (Hernoux et al., 2015; Arntz et al., 2021a), participants can be exposed to various human-like gestures portrayed by the robot from different perspectives independent of their actual height. Apart from the presentation of the stimulus material, it is necessary to discuss the execution of the gestures. Restrictions in the kinematics of the robot made slight alterations necessary combined with the absence of some anatomical characteristics such as the “hips” of the robot. While not completely accurate, the gestures followed the same trajectory as their human counterpart, aiming for an authentic representation of these gestures. In addition to the mere observation of these human-like gestures, participants should execute a shared task collaboration scenario in conjunction with the robot to further emphasize the context of these gestures. This circumstance addresses another major limitation of the presented study. Considering that participants merely observed the gestures of the robot through the video-based stimulus material, it can be argued that the stimulus material might not induce the same reaction compared to a study setup where people are confronted with the real robot. However, apart from the COVID-19-related restrictions for the execution of lab studies, it can be argued that the stimulus material ensured the comparability of the self-reported answers because every participant was exactly exposed to the same gestures presented from the same perspective. Additional artifacts such as technical difficulties that might occur by exposing participants to the real robots were therefore avoided. Nonetheless, a future study should refine the approach in a lab study as mentioned before. Another limitation is the neglect of further demographic-related variables apart from age and gender that prevent further contextualization of the data based on the participants’ background. The limitation lies in the inability to rate the sample composition for its applicability to a general population. Since this is not possible the participants of this study must be considered as a convenience sample. An additional limitation regarding the questionnaire is the omission of the full Godspeed scale. While this resulted in low reliability (cf. Section 3.2), the reduction in sub-scales was done to prevent the questionnaire to become too extensive. Since the presented material was already lengthy and a further elongation of the questionnaire might have discouraged participants to complete the questionnaire.

## 6 Conclusion

The usage of gestures in collaboration robots, especially representations capable of mimicking human-like gestures such as the Yumi IRB 14000 dual-arm robot, is a promising channel to convey situational information and elevate collaboration effectiveness. However, body language is also up for interpretation, as it does not contain a direct message from the sending entity to the receiving entity. Especially in industrial robots, where body language that is found in humans can not exactly be recreated compared to distinctly designed social robots, it is important to explore the individual perception of the gestures to evaluate their capability to elicit certain impressions of the robot onto the operator. The research presented here marks the first foray into the vast library of human body language expressions that can be translated onto collaborative robots. Upcoming studies should incorporate more gestures that are associated with different attributes apart from dominance to explore their effect on people’s perception of the robot. Furthermore, future studies are recommended to embed these gestures into a collaboration procedure with the robot, to investigate the direct ramifications of the usage of these gestures on the collaborative relationship between both parties.

## Data Availability

The raw data supporting the conclusion of this article will be made available by the authors, without undue reservation.
